# You can have your cake and eat it too: Ectopic expression of *COLD-REGULATED* genes reshapes the salicylic acid–mediated growth-defense tradeoff

**DOI:** 10.1093/plcell/koae230

**Published:** 2024-08-14

**Authors:** Leiyun Yang

**Affiliations:** Assistant Features Editor, The Plant Cell, American Society of Plant Biologists; Department of Plant Pathology, College of Plant Protection, Nanjing Agricultural University, Key Laboratory of Integrated Management of Crop Diseases and Pests, Ministry of Education, Nanjing, 210095, China; The Key Laboratory of Plant Immunity, Nanjing Agricultural University, Nanjing, 210095, China

One of my favorite sayings is “You can’t have your cake and eat it too.” This adage reflects the numerous choices we face daily, often necessitating sacrifices in decision-making. Similarly, plants maintain a growth-defense balance under normal conditions. However, this balance shifts in favor of defense upon pathogen infection, ultimately compromising growth. This phenomenon, known as the growth-defense tradeoff, is also observed in autoimmune mutants, which exhibit dwarfism and constitutive activation of salicylic acid (SA)-mediated defense responses (Yang et al. 2022). Given the strong negative correlation between growth and immunity, autoimmune mutants serve as valuable tools for studying the SA-mediated growth-defense tradeoff. Nonetheless, autoimmune mutants often have pleiotropic phenotypes due to the complex function of the causal genes, complicating the study of this phenomenon.

To address this, **María Ortega and colleagues (Ortega et al. 2024)** expressed a bacterial bifunctional SA synthase *Irp9* (Xue et al. 2013) in Arabidopsis (*A. thaliana*) and obtained transgenic lines with varying SA levels. Among these lines, F24, F31, F36, and F51 exhibited progressively higher SA levels than the wild type (WT) and were designated as high-SA (hiSA) lines. The hiSA lines displayed noticeable growth defects, including reduced rosette and root biomass, accelerated senescence, early flowering, and decreased seed yield (Fig.) Notably, the growth defects in these lines were inversely correlated with their total SA levels, with F51 showing the most severe phenotypes, followed by F36, F31, and F24. These results suggest an inhibitory role of SA on plant growth. After infection with the bacterial pathogen *Pseudomonas syringae* pv. tomato strain DC3000, the hiSA lines exhibited significantly less bacterial growth than the WT, as expected. Taken together, these data demonstrate that SA has inhibitory effects on plant growth while promoting plant disease resistance.

**Figure.**
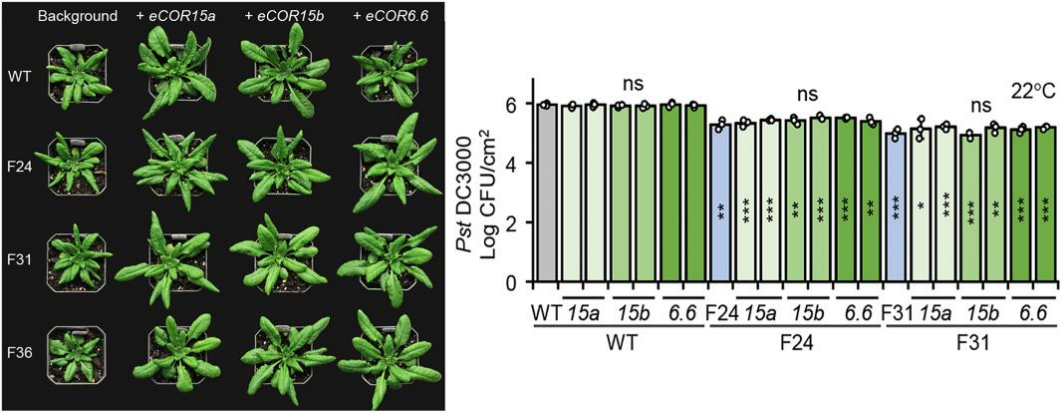
The hiSA lines (F24, F31, and F36) exhibited compromised growth due to high SA accumulation and constitutive activation of defense responses. Ectopic expression of *COR* genes rescued the growth penalty without interfering with the enhanced disease resistance of the hiSA lines. For each line, no statistical significance (ns) was found against the respective background. Statistical differences of F24 and F31 compared with WT are indicated by asterisks inside the bar (***P < 0.001; **P < 0.01; *P < 0.05). Adapted from Ortega et al. (2024) Figure 6.

Transcriptomic analyses of the WT, hiSA lines, and an SA-deficient NahG (SA hydroxylase) transgenic line revealed a positive correlation between the number of differentially expressed genes (DEGs) in the transgenic lines compared with WT, with F51 and NahG having the highest number and sharing 471 DEGs. These 471 DEGs were clustered into SA-induced and SA-repressed groups. SA-induced DEGs were significantly enriched in gene ontology terms “systemic acquired resistance,” “response to SA,” and various immune signaling pathways. In contrast, SA-repressed DEGs were associated with “cold acclimation” and “response to water deprivation.” Of note, members of 4 *COLD-REGULATED* (*COR*) gene families known to be induced by cold (Thomashow 1999) were highly represented, suggesting an inhibitory effect of SA on *COR* gene expression. Consistently, F24, WT, and NahG plants all exhibited decreased growth as the temperature shifted from 26 °C to 16 °C, and the growth penalty was positively correlated with SA levels.

Based on these findings, the authors hypothesized that constitutive expression of *COR* genes in hiSA lines would compensate for the repression of *COR* by SA, thereby alleviating the resulting growth penalty. To test this, the authors generated transgenic overexpression lines of *COR15* in hiSA lines. Overexpression of *CORs* rescued the growth defects of hiSA lines without affecting their high SA levels and enhanced disease resistance at both normal and low temperatures. These data suggest that ectopic *COR* expression rescues the growth inhibition caused by SA at both temperatures without interfering with SA-mediated disease resistance.

This study demonstrates that SA inhibits plant growth by repressing *COR* gene expression and highlights how the transcriptional rewiring of *COR* genes can reshape SA-mediated growth-defense tradeoff. It supports the notion that the growth-defense tradeoff may be caused by interference between different defense and growth signaling pathways. These findings open a new avenue for genetically engineering more resilient crops without incurring a growth penalty.
